# Importance of A-loop complementarity with tRNA^His ^anticodon for continued selection of tRNA^His ^as the HIV reverse transcription primer

**DOI:** 10.1186/1743-422X-4-4

**Published:** 2007-01-10

**Authors:** Na Ni, Wenqin Xu, Casey D Morrow

**Affiliations:** 1 Department of Cell Biology, University of Alabama at Birmingham, Birmingham, AL 35294-0024, USA

## Abstract

**Background:**

Human immunodeficiency virus (HIV-1) preferentially selects tRNA^Lys,3 ^as the primer for reverse transcription. HIV-1 can be forced to select alternative tRNAs through mutation in the primer-binding site (PBS) and a region upstream of the PBS designated as the A-loop. Alteration of the PBS and A-loop to be complementary to the 3' terminal nucleotides and anticodon of tRNA^His ^results in HIV-1 that can stably utilize this tRNA for replication.

**Results:**

In the current study, we have investigated the effect that mutations within the A-loop have on the stability of HIV-1 with a PBS complementary to tRNA^His^. For these studies, we have altered the A-loop to be complementary to tRNA^Met^, tRNA^Gln^, tRNA^Ile^, tRNA^Thr ^and tRNA^Ser^. All substitutions of the A-loops with the PBS complementary to tRNA^His ^resulted in a reduction of infectious virus obtained following transfection of proviral genomes in the 293T cells. Virus replication in SupT1 cells was also impaired as a result of the alteration of the A-loop. Viruses with the A-loop complementary to tRNA^Lys,3 ^and tRNA^Ser ^reverted to utilize tRNA^Lys,3 ^following *in vitro *replication. In contrast, viruses with the A-loop complementary to the other tRNAs remained stable and continued to use tRNA^His^. RNA modeling of the stem-loop structure revealed that nucleotides were displayed on the loop region that could potentially interact with the anticodon of tRNA^His^. To further explore the effects of the A-loop mutations on virus replication, the A-loops complementary to tRNA^Ser ^or tRNA^His ^were cloned into the wild type genome with the PBS complementary to tRNA^Lys,3^. Transfection of proviral genomes which contained the wild type PBS and A-loops complementary to tRNA^Ser ^or tRNA^His ^into 293 T cells did not impact on the production of viruses as measured by p24 antigen ELISA. However, viruses with the A-loop complementary to tRNA^His ^had greatly reduced infectivity and replicated poorly in SupT1 compared to the wild type or viruses with the A-loop complementary to tRNA^Ser^.

**Conclusion:**

These studies demonstrate that complementarity of A-loop region with the anticodon of tRNA^His ^has a pronounced effect on the capacity of HIV-1 to utilize tRNA^His ^as the primer for reverse transcription. Complementarity between A-loop and anticodon of the tRNA then is important for the selection of the tRNA primer used for reverse transcription.

## Background

The hallmark of retrovirus replication is the process by which the RNA genome is converted to a DNA intermediate prior to integration into the host cell chromosome. This process, termed reverse transcription is catalyzed by a virally encoded enzyme reverse transcriptase [1,2]. A cellular tRNA is captured by retroviruses for use as the primer for the initiation of reverse transcription. The 3' terminal 18-nucleotides of the tRNA is complementary to an 18-nucleotide region in the viral RNA genome designated as the primer-binding site (PBS) [3-5]. The reverse transcriptase uses the tRNA bound to the PBS as the primer to copy the viral genome. During reverse transcription, the reverse transcriptase copies the tRNA primer to regenerate the PBS. Thus, the PBS of integrated proviruses is complementary to the tRNA primer used for initiation [6-8].

Human immunodeficiency virus (HIV-1) preferentially selects tRNA^Lys,3 ^as the primer for replication [9-12]. Previous studies have shown that alteration of the PBS to be complementary to alternative tRNAs, allows HIV-1 to transiently utilize these tRNAs for replication [13-15]. However, HIV-1 with the PBS complementary to these alternative tRNAs revert following *in vitro *culture to utilize tRNA^Lys,3^. Previous studies from this lab have attempted to force HIV-1 to utilize alternative tRNAs for replication [16-20]. To achieve this, mutations have been made in an upstream region, designated as the A-loop. Previous studies using both chemical and enzymatic analysis have shown that the A-loop interacts with the anticodon region of tRNA^Lys,3 ^in the initiation complex [21]. Alteration of the A-loop region to be complementary to certain tRNA molecules along with mutations in the PBS allow HIV-1 to utilize certain alternative tRNAs as primer for replication. Using this approach, we have generated viruses which can utilize tRNA^Lys1,2^, tRNA^His^, tRNA^Glu^, tRNA^Met ^and more recently tRNA^Thr ^[16-19,22,23]. HIV-1 with alterations in the A-loop and PBS to be complementary to tRNA^Ile^, tRNA^Ser ^or tRNA^Gln ^though were not stable and rapidly reverted back to utilize tRNA^Lys,3 ^following *in vitro *replication [20,23,24]. The reason for the preference of HIV-1 for a certain tRNAs that can be selected and utilized as primers for replication is unknown. It is possible that primer selection occurs from an intracellular pool of tRNAs. In support of this idea, previous studies have shown a link between primer selection and viral translation [25,26].

One of the more thoroughly characterized HIV-1 that utilizes alternative tRNA is a virus that has been engineered to replicate using tRNA^His ^[16,17]. Previous studies from our laboratory have shown that alteration of the A-loop and PBS to be complementary to tRNA^His ^results in a virus that can stably utilize this tRNA for *in vitro *replication. Analysis of virus that had undergone extensive *in vitro *replication revealed a RNA stem-loop structure in which the nucleotides complementary to the anticodon of tRNA^His ^were displayed on the loop region. A previous study has shown that this RNA stem loop structure has the potential to interact with tRNA^His ^*in vitro *[27].

In the current study, we have further explored the specificity of the A-loop in the primer selection process. Since RNA structure of the A-loop is probably important, we have used A-loop regions from viruses that have been engineered to stably utilize alternative tRNAs (tRNA^Met ^and tRNA^Thr^). We have also used A-loop regions that are complementary to the anticodon of tRNA^Ile^, or tRNA^Ser ^or tRNA^Gln ^which are not stably used by HIV-1. The A-loop regions thus display nucleotides complementary to the different tRNAs on the loop of an RNA stem-loop. Analysis of the replication and stability of the PBS revealed that certain A-loops effectively substitute for the A-loop complementary to the anticodon of tRNA^His ^to allow the virus to stably utilize this tRNA for replication. The presence of nucleotides that can interact with the anti-codon of tRNA^His ^correlates with the stability of these viruses following *in vitro *replication. Alteration of the A-loop region complementary to the anticodon of tRNA^His ^can also impact on the replication of the wild type virus. The results of these studies demonstrate that the complementarity of the A-loop region with the anticodon region of tRNA^His ^is important for the stable selection and use of this tRNA as the primer for reverse transcription.

## Results

### Construction of HIV-1 with A-loop mutations and PBS complementarity to tRNA^His^

**Figure 1:**
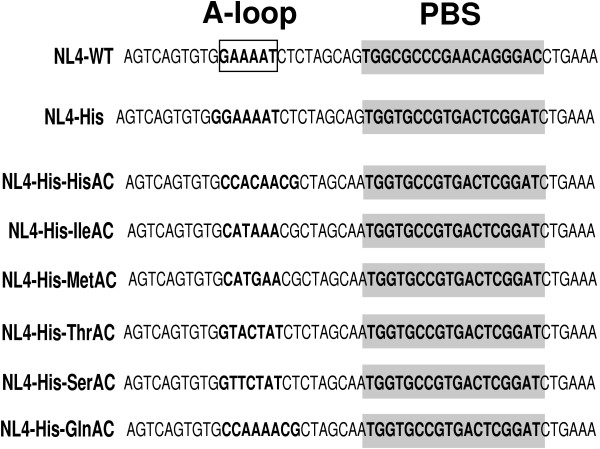
**HIV-1 proviral genomes with mutated A-loop and PBS**. The A-loop (boxed) and PBS (shaded) were altered in the NL4 proviral clone to be complementary to tRNA^His^, tRNA^Ile^, tRNA^Met^, tRNA^Thr^, tRNA^Ser ^or tRNA^Gln^. The NL4-WT contains a PBS and A-loop complementary to the 3' terminal 18 nucleotides and the anticodon of tRNA^Lys,3^. The NL4-His contains a PBS complementary to tRNA^His ^but the A-loop is complementary to the anticodon region of tRNA^Lys,3^. The remaining proviral clones contain a PBS complementary to tRNA^His ^with additional mutations in the A-loop to be complementary to the anticodon of the designated tRNA (e.g. NL4-His-HisAC).

In previous studies, we have described HIV-1 proviral genome in which the PBS has been altered to be complementary to tRNA^His ^that maintains the A-loop complementary to tRNA^Lys,3^(NL4-His) [16,17]. We have also previously described proviral genomes in which both the A-loop and PBS have been made complementary to the anticodon and 3' terminal nucleotides at tRNA^His ^(NL4-His-HisAC). Previous studies have shown that this virus stably maintains a PBS complementary to tRNA^His ^following replication in SupT1 cells. In the current study, we have made use of the A-loop regions that are complementary to tRNA^Met^, tRNA^Ile^, tRNA^Gln^, tRNA^Ser ^and tRNA^Thr^. These A-loop regions were cloned into proviral genomes in which the PBS was complementary to tRNA^His^. The resulting viruses were named NL4-His-MetAC, NL4-His-IleAC, NL4-His-GlnAC, NL4-His-SerAC and NL4-His-ThrAC (Figure 1).

We first analyzed the production of infectious virus following transfection of the proviral genomes into 293T cells. In general, we found that the amount of virus produced as determined p24 antigen from the transfection supernatant was comparable for all of the mutants and similar to that of the wild type virus (NL4-WT). Thus, alteration of the A-loop and PBS did not effect virus production as measured by p24 antigen. However, alteration of the PBS resulted in a reduction in the production of infectious virus, as determined by the JC53-BL assay, to levels that were approximately 20% that of the wild type virus. This result is consistent with previous studies from our laboratory which have shown that alteration of the PBS generally results in a reduction production of infectious virus, but not viral proteins, as compared to the wild type virus.

**Figure 2:**
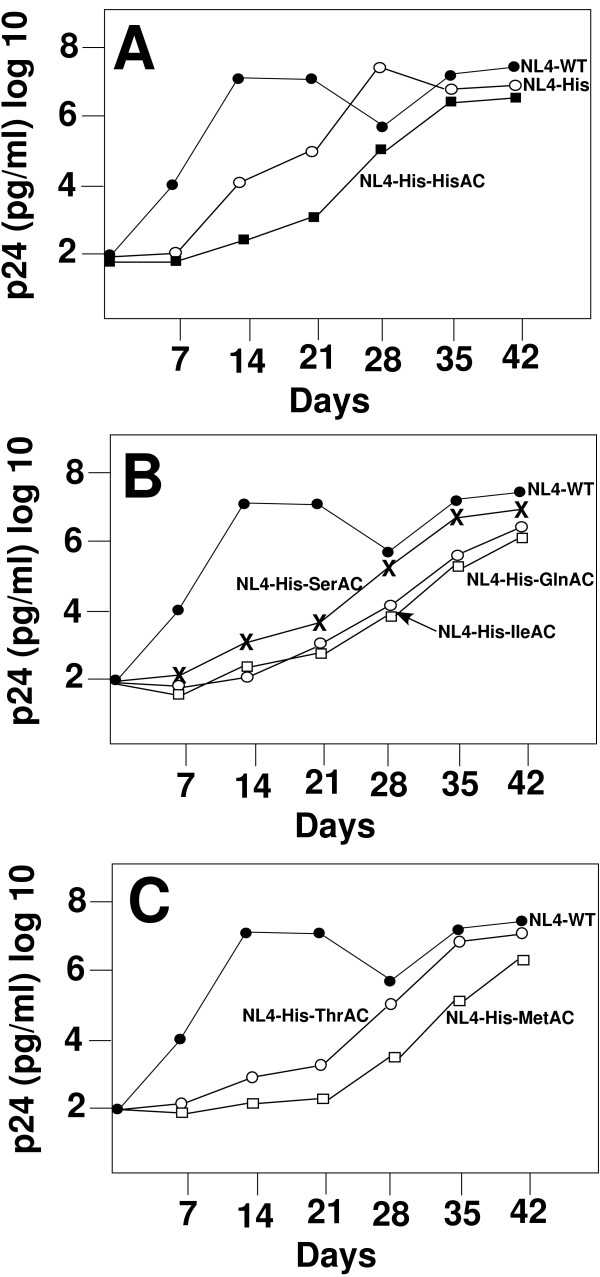
**Replication of HIV-1 with wild type and altered U5-PBS in SupT1 cells**. **Panel A**. Replication of HIV (NL4-WT) and HIV which contain U5-PBS complementary to tRNA^His^. Infections were initiated with equal amounts of infectious virus as determined by the JC53-BL assay. Virus replication in SupT1 cells was monitored over 42 days of culture. The amount of virus produced was analyzed by p24 ELISA. The identity of the viruses are as marked. **Panel B. **Comparison of the replication of the wild type virus with mutant viruses containing PBS complementary to tRNA^His ^with different A-loop regions. The identity of the viruses are as marked. **Panel C. **Comparison of the replication of the wild type virus with HIV-1 genomes with the PBS complementary to tRNA^His ^with different A-loop regions. The identity of the viruses are as marked.

To determine the effects of the A-loop mutations on the replication of virus, we analyzed the replication following culture in SupT1 cells. Infections were established with the same amounts of infectious virus and was monitored by the production of viral proteins (p24) in the supernatant using the p24 antigen ELISA capture assay. Analysis of the replication of SupT1 culture infected with the wild type (NL4-WT) virus revealed a rapid rise in the production of p24 antigen in the cultures that peaked at approximately 14 days and was maintained throughout the duration of the infection (42 days). The virus in which the PBS was altered to be complementary to tRNA^His ^(NL4-His) exhibited a delay in the increase of replication, but eventually reached a peak level similar to the wild type virus at Day 28 of culture. In contrast, virus in which the PBS and A-loop were complementary to tRNA^His ^(NL4-His-HisAC), exhibited a substantial delay in replication of virus reaching a peak only at Day 35 of culture (Figure 2A). Consistent with our previous studies, analysis of the PBS of these viruses revealed that the PBS of NL4-His had reverted back to utilize tRNA^Lys,3^; this reversion occurred by approximately Day 28 post initiation of culture when we observed the rise in p24 antigen in the culture supernatant. In contrast, NL4-His-HisAC retained a PBS complementary to tRNA^His ^for the duration of the culture.

We next compared the replication of viruses in which the PBS was complementary to tRNA^His ^with different A-loop combinations. In the first of experiments, we analyzed the replication of NL4-His-SerAC, NL4-His-IleAC and NL4-His-GlnAC. Previous studies have shown that viruses with the U5-PBS complementary to these tRNAs (tRNA^Ser^, tRNA^Ile ^and tRNA^Gln^) were unstable and reverted to tRNA^Lys,3 ^during culture [24,28]. The replication of NL4-His-SerAC was delayed compared to that of the wild type virus, eventually reaching a peak level at Day 35 post initiation of culture. The viruses derived from NL4-His-GlnAC and NL4-His-IleAC also exhibited a delay in replication reaching a peak at a similar time approximately 42 days post initiation of culture (Figure 2B). Analysis of the PBS of these viruses revealed that NL4-His-SerAC had reverted to utilize tRNA^Lys,3^. In contrast, viruses derived from NL4-His-GlnAC and NL4-His-IleAC retained the PBS complementary to tRNA^His ^for the duration of the culture. Finally, we analyzed the replication of viruses in which the A-loop had been mutated to be complementary to tRNA^Met ^or tRNA^Thr^; previous studies have shown that U5-PBS complementary to these tRNAs remain stable following *in vitro *replication [18]. All of these viruses exhibited a delayed replication compared to the wild type virus. It was only during the end of the culture period (approximately 30–42 days) that these viruses reached peak levels of p24 antigen in the culture (Figure 2C). However, analysis of the PBS of these viruses revealed that all had retained the PBS complementary to tRNA^His^, indicating a stable use of this tRNA as the primer for replication.

**Figure 3:**
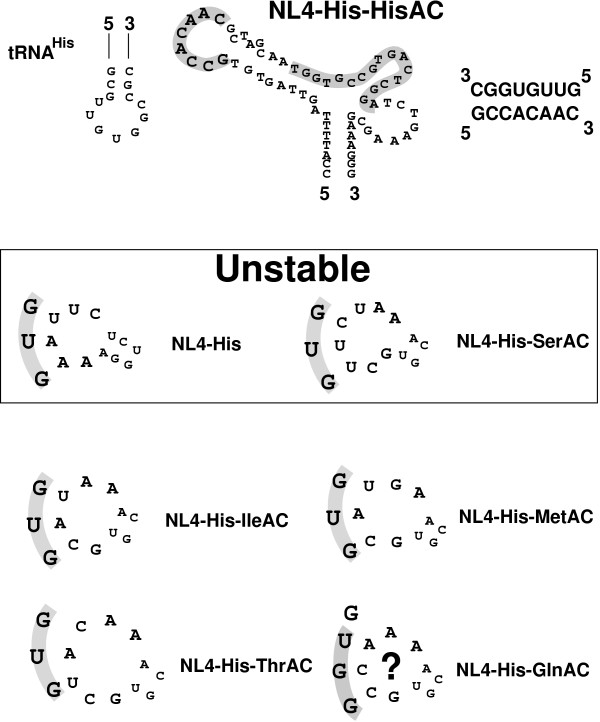
**Potential interaction between the anticodon region of tRNA^His ^and HIV-1 proviral genomes with mutated A-loop regions**. The A-loop PBS region of NL4-His-HisAC depicting the nucleotides displayed on the loop of an RNA stem loop that are complementary to the anticodon of tRNA^His^. In the case of NL4-His-HisAC, complete complementarity is noted consisting of eight consecutive nucleotides. Viruses in which the PBS complementary to tRNA^His ^were unstable following *in vitro *replication included those derived from NL4-His and NL4-His-SerAC (bolded, unstable). Interactions between the anticodon of tRNA^His ^and NL4-His or NL4-His-SerAC consisted of two potential base pair interactions (shaded). In contrast, interaction between the anticodon of tRNA^His ^and viruses which stably maintained a PBS complementary to tRNA^His ^(NL4-His-IleAC, NL4-His-MetAC or NL4-His-ThrAC) revealed three potential base pair interactions (shaded). The virus NL4-GlnAC was also stable following *in vitro *replication. A three-nucleotide interaction could be postulated (in shaded regions) consist of two of the three nucleotides in the anticodon of tRNA^His^. This putative interaction is denoted with a question mark.

The results from the growth and stability of PBS with the different A-loop regions were somewhat surprising. Two viruses, NL4-His and NL4-His-SerAC grew well but the PBS was unstable and reverted back to utilize tRNA^Lys,3^. In contrast, the viruses with a PBS complementary to tRNA^His ^containing different A-loops grew slower but maintained a PBS complementary to tRNA^His^. Particularly surprising in this group was the virus NL4-His-IleAC and NL4-His-GlnAC which grew slowly and maintained a PBS complementary to tRNA^His^, since previous studies have shown that viruses in which both the PBS and A-loop were complementary to tRNA^Ile ^or tRNA^Gln ^rapidly reverted back to utilize tRNA^Lys,3 ^following *in vitro *replication [18]. We had suspected that NL4-His-IleAC and NL4-HisGlnAC would have phenotypes similar to that of NL4-His-SerAC and revert back to utilize tRNA^Lys,3 ^following replication. To gain further insights into this result, we analyzed the potential RNA structure of the U5 A-loop regions using *mfold*. The *mfold *of the U5-PBS from NL4 revealed a RNA stem-loop structure in which the A-loop region was displayed on the loop. Similarly, the *mfold *of NL4-His-HisAC also revealed a RNA stem-loop structure in which the anticodons complementary to the tRNA^His ^were displayed on the loop of the RNA stem-loop (Figure 3). We next compared the *mfolds *of NL4-His-SerAC and NL4-His-IleAC. In the case of NL4-His-SerAC, as expected a nucleotide sequence was displayed on the loop that was complementary to tRNA^Ser^. Similarly, the *mfold *of NL4-His-IleAC also displayed a nucleotide sequence complementary to the anticodon of tRNA^Ile^. However, we found that the nucleotides displayed on the loop of NL4-His-IleAC could also base pair with the anticodon region of tRNA^His^, which was not the case for the loop region from NL4-His-SerAC. A similar analysis of the additional viruses revealed the presence in the loop region of nucleotides that could base pair with tRNA^His^; this also included NL4-His-GlnAC, where nucleotides within the anticodon loop (not the three nucleotides of the anticodon) could base pair. Thus, the stability of the PBS complementary to tRNA^His ^correlated, in part, with the presence of nucleotides in the loop complementary to the anticodon of tRNA^His^.

### Effect of A-loop alteration on replication of wild type virus

**Figure 4:**
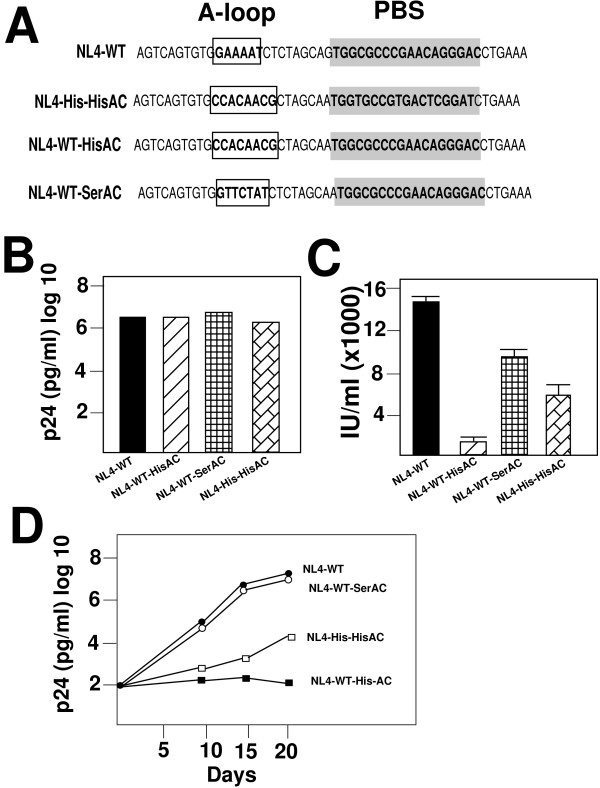
**Analysis of HIV-1 viruses with a wild type PBS complementary to tRNA^Lys,3 ^with mutated A-loop regions**. **Panel A. **HIV-1 proviral genomes with the wild type PBS and A-loop (NL4-WT), wild type PBS and A-loop regions mutated to be complementary to tRNA^His ^(NL4-WT-HisAC) or tRNA^Ser ^(NL4-WT-SerAC). Additional proviral constructs included HIV genomes with a PBS and A-loop region complementary to tRNA^His ^(NL4-His-HisAC). **Panel B. **Production of virus following transfection of wild type and mutant proviral genomes. 293T cells were transfected with equal amounts of proviral genomes and the amount of virus released into the supernatant was determined by p24 antigen capture ELISA. All viruses had similar amounts of p24 antigen into the culture supernatant (representative of three transfections). **Panel C. **Infectious virus was produced following transfection. Viruses were transfected with equal amounts of proviral genomes and the amount of infectious virus determined by the JC53-BL assay. Results are the average of the three determinations of infectivity in JC53-BL cells. **Panel D. **Replication of HIV-1 proviral genomes in SupT1 cells. Infections were established with equal amounts of infectious virus as determined by the JC53-BL assay. The amount of infectious virus was determined by the p24 antigen capture assay. The identity of the viruses are as marked.

To further analyze the effects of the alteration of the A-loop region on virus replication, we constructed HIV-1 proviruses with a PBS complementary to tRNA^Lys,3 ^and an A-loop complementary to either tRNA^Ser ^or tRNA^His ^(Figure 4A). We first analyzed the production of viruses following transfection into 293T cells (Figure 4B). Viruses derived from transfection of the wild type (NL4-WT) and viruses with the A-loop complementary to tRNA^Ser ^(NL4-WT-SerAC) or tRNA^His ^(NL4-WT-HisAC) all gave similar amounts of p24 antigen following transfection. Alteration of the A-loop region then did not impact substantially on the production of viral proteins, consistent with our previous results. We next analyzed the supernatants for infectious virus using the JC53-BL assay. Viruses derived from NL4-WT-SerAC were slightly less infectious than the wild type virus. In contrast, we observed substantially reduced production of infectious virus following transfection of NL4-WT-HisAC, indicating a pronounced effect of the alteration of the A-loop to be complementary to tRNA^His^. Interestingly, analysis of the infectious virus produced from transfection of NL4-His-HisAC revealed the amount was approximately 4 – 5 times greater than that obtained from NL4-HisAC, indicating that the preference for HIV-1 to select tRNA^His ^can be strongly influenced by the inclusion of the A-loop complementary to tRNA^His ^(Figure 4C).

We next compared the replication of theses viruses in SupT1 cells (Figure 4D). In the case of the wild type virus (NL4-WT), a rapid increase in the production of infectious virus, peaking at approximately Day 14 to 21-post initiation of culture. The virus derived from NL4-SerAC gave a similar replication profile peaking again at Day 14 to 21. In contrast, virus derived from NL4-HisAC was poorly infectious during the culture period examined barely producing enough virus to be detected using the p24 ELISA. In fact, replication of NL4-His-HisAC was approximately 100 fold greater than NL4-HisAC (as determined by p24 antigen in the culture supernatant), indicating the A-loop modification with change in the PBS to be complementary to tRNA^His ^facilitated selection and use of tRNA^His ^as the replication primer.

## Discussion

Previous studies from this laboratory have utilized a genetic approach to understand the mechanism of primer selection by HIV-1 [16-20,22]. Alteration of both the A-loop region and PBS to be complementary to several different tRNAs resulted in HIV-1 having the capacity to stably utilize these tRNAs for replication. Viruses which can utilize tRNA^His^, tRNA^Met^, or tRNA^Thr ^have been previously described [16-18,23]. All of these viruses maintain the PBS complementary to the cognate tRNA, although their replication is diminished compared to the wild type. In contrast, the viruses in which PBS and A-loop were complementary to tRNA^Ile^, tRNA^Ser^, or tRNA^Gln ^did not utilize these tRNAs following *in vitro *culture, and rapidly reverted to utilize tRNA^Lys,3 ^[20,23,24]. It was not clear though, from our previous studies if the nucleotide sequence within the A-loop region and the RNA structure of this A-loop region were important for the virus to maintain a PBS to an alternative tRNA. The results of our current study highlight the importance of the nucleotide sequence displayed on the A-loop region for the unique selection of tRNA^His ^as the primer for replication. Only those viruses in which the A-loop region contained nucleotides that could interact with the anticodon region of tRNA^His ^stably maintained the PBS complementary to tRNA^His ^following replication. Indeed, even viruses NL4-His-IleAC and NL4-His-GlnAC stably maintained a PBS complementary to tRNA^His ^following *in vitro *replication. Viruses in which the PBS and A-loop region were complementary to tRNA^Ile ^or tRNA^Gln ^though rapidly reverted to utilize tRNA^Lys,3 ^following *in vitro *replication [20,23,24]. One explanation for this discrepancy is that the availability of tRNA^His^, tRNA^Ile ^and tRNA^Gln ^could differ for selection as primers for HIV-1 reverse transcription. We would expect that if tRNA^Ile ^or tRNA^Gln ^were available for primer selection, it would compete with tRNA^His ^for interaction with the A-loop region which could potentially compromise the stability of the PBS following *in vitro *replication. The stability of the A-loop regions complementary to tRNA^Met ^and tRNA^Thr ^that allow the virus to stably utilize tRNA^His ^might mean that tRNA^His ^has a greater intracellular availability for primer selection. This explanation will also account for the results obtained with the virus NL4-His-SerAC, which contained nucleotides displayed on the loop region that are complementary to the anticodon of tRNA^Ser^. This virus rapidly reverted to utilize tRNA^Lys,3 ^following *in vitro *replication. Without tRNA^Ser ^available to interact with the A-loop, and because the nucleotides in the A-loop of NL4-His-SerAC are not complementary to the anticodon of tRNA^His^, tRNA^Lys,3 ^would be favored for capture, leading to the conversion of the PBS to be complementary to tRNA^Lys,3^.

The results of our studies support the idea that A-loop region plays an important role in the selection of tRNA^His ^as an alternative primer for HIV-1 replication [16,17]. Previous studies have utilized *in vitro *systems to demonstrate that the A-loop region does interact with the anticodon of tRNA^His ^[27]. The results of our current studies again support that this interaction is important for the continued selection of tRNA^His ^as the primer for reverse transcription. A surprising result from our studies was the impact of the A-loop region complementary to tRNA^His ^on the replication of the wild type virus with the PBS complementary to tRNA^Lys,3 ^(NL4-WT-HisAC). In fact, NL4-WT-HisAC replicated poorly compared to the wild type virus or NL4-WT-Ser which contained the A-loop region complementary to tRNA^Ser^. This inhibition of replication was not due to production of virus as demonstrated following transfection of proviral genomes in the 293T cells where we recovered similar amounts of p24 antigen in the culture supernatants. Rather, the major effect was on the production of infectious virus as determined both by the JC53-BL assay and from analysis of virus replication studies in SupT1 cells. How the A-loop complementary to the anti-codon would inhibit replication of the virus with a PBS complementary to tRNA^Lys,3 ^is unclear. One possibility could be that because of the availability of tRNA^His^, the A-loop attracts tRNA^His ^to the U5-PBS. The presence of both tRNA^His ^and tRNA^Lys,3 ^with the U5-PBS could interfere with primer selection, accounting for the lower production of infectious virus.

## Conclusion

In the current study, we have further investigated the mechanism of HIV-1 primer selection using a unique virus which has been engineered to use tRNA^His ^as the primer for replication. For HIV-1 to select tRNA^His ^as the primer, previous studies have found that additional mutations were required in the A-loop region upstream of the PBS to be complementary to the anticodon of tRNA^His ^[16,17]. Viruses in which the A-loop region contains nucleotides complementary to the anticodon of tRNA^His ^stably maintained the PBS complementary to tRNA^His ^following *in vitro *replication. In contrast, viruses which the A-loop contained nucleotides that could not interact with the anticodon of tRNA^His ^(NL4-His-SerAC or NL4-His) reverted to utilize tRNA^Lys,3 ^following a short-term *in vitro *culture. Substitution of the A-loop in the wild type genome to be complementary to the anticodon of tRNA^His ^rather than tRNA^Lys,3 ^had a profound impact on virus replication.

Our results are consistent with the idea that there is an interaction between the tRNA and A-loop region and PBS could facilitate the selection of the primer. If the A-loop region is complementary to alternative tRNAs that are available (e.g. tRNA^His^), this interaction could interfere with the efficient selection of tRNA^Lys,3^. A previous study described several alternative RNA structures in the 5' NTR that could function as a riboswitch for dimerization and encapsidation [29]. When considered in the context of the results presented in this study, it is possible that the RNA structures could be involved in the selection of the tRNA primer through interaction with the A-loop and PBS. Many riboswitch elements have been identified in the 5' NTR of bacterial RNAs including T-box RNAs which interact with tRNAs [30-33]. It is possible that the process of primer selection/capture could involve a riboswitch like mechanism in which the A-loop-PBS co-ordinates the capture of available tRNAs, such as tRNA^His^, tRNA^Met^, tRNA^Thr ^and of course tRNA^Lys,3^. Additional experiments will be needed to delineate the important features of the interaction between the U5-PBS and tRNA that are involved in primer selection.

## Materials and methods

### Construction of mutant proviral genomes

All mutations were made in pUC119PBS or derivatives using the Quick change II site-directed mutagenesis kit (Stratagene, LaJolla, CA). The pUCHis-SerAC, pUCHis-ThrAC, pUCHis-IleAC, pUCHis-MetAC, pUCHis-GlnAC are the mutants with PBS complementary to tRNA^His ^while A-loop complementary to the anticodon of tRNA^Ser^, tRNA^Thr^, tRNA^Ile^, tRNA^Met ^and tRNA^Gln ^respectively. For all these pUCHis mutants, the modifications in A-Loop were made with pUC119HisAC as the template. In pUC119HisAC, both PBS and A-Loop are pared with tRNA^His^. The primers for pUC119His-SerAC were 5'-GACCCTTTTAGTCAGTGTGCTTCTAACGCTAGCAAT GGTGC-3' (sense) and 5'-GCACCATTGCTAGCGTTAGAAGCACACTGACTAAAA GGGTC-3' (anti-sense); the primers for pUC119His-ThrAC were 5'-GACCCTTTTAGT CAGTGTGCTACAAACGCTAGCAATGGTGC-3' (sense) and 5'-GCACCATTGCTA GCGTTTGTAGCACACTGACTAAAAGGGTC-3' (anti-sense); the primers for pUC119His-IleAC were 5'-GACCCTTTTAGTCAGTGTGCATAAACGCTAGCAATG GTGCC-3' (sense) and 5'-GGCACCATTGCTAGCGTTTATGCACACTGACTAAAA GGGTC-3' (anti-sense); the primers for pUC119His-MetAC were 5'-GACCCTTTTAG TCAGTGTGCATGAACGCTAGCAATGGTGCC-3' (sense) and 5'-GGCACCATTGC TAGCGTTCATGCACACTGACTAAAAGGGTC-3' (anti-sense) and the primers for pUC119His-GlnAC were 5'-GACCCTTTTAGTCAGTGTGCCAAAACGCTAGCAAT GGTGC-3' (sense) and 5'-GCACCATTGCTAGCGTTTTGGCACACTGACTAAAAG GGTC-3' (anti-sense). To construct the mutants with wild type PBS and altered A-loop regions, the starting plasmid was pUC119PBS. To construct HIV-1 with the A-loop to be complementary to tRNA^His ^with PBS complementary to tRNA^Lys,3^, the three additional nucleotides 628, 635, 654 were changed to the G, A and C, because these three mutations have been shown to facilitate the usage of tRNA^His ^together with A-loop mutations [17,27]. The site-directed mutagenesis was carried out on the plasmid pUC-119 vector that has wild type PBS. The synthetic oligonucleotides primers to construct pUC-WT-HisAC were 5'-GTCAGTGTGGAAAATCGCTAGCAATGGCGCCCGAACAGGGACCTGA AAGCGAAAGGGAAAC-3' (sense) and 5'-GTTTCCCTTTCGCTTTCAGGTCCCTGT TCGGGCGCCATTGCTAGCGATTTTCCACACTGAC-3' (antisense). Following the mutagenesis, the clones were confirmed by DNA sequencing. All the pUC119 mutants were digested with *HpaI *and *BssHII *and inserted into *SmaI *and *BssHII *sites of NL4-3 HIV-1 proviral plasmid. The resulting NL4-3 mutants were verified by the automated DNA sequencing.

### Tissue culture

The 293T cells were maintained in Dulbecco's modified Eagle's medium (DMEM) (Cellgro, Hemdon, VA) plus 10% fetal bovine serum (FBS) (Hyclone, Logan UT) and 1% antibiotic-antimycotic (Bibco BRL, Rockville, MD). SupT1 cells were maintained in RPMI 1640 (Cellgro, Hemdon, VA), supplemented with 15% FBS and 1% antibiotic-antimycotic.

### DNA transfections

Transfections were performed according to the protocol of Fugene 6 transfection Reagent (Roche Molecular Biochemicals, Indianapolis, IN). 3 ul Fugene reagent was added to 100 ul of DMEM without FBS, the 2 ug of proviral plasmid DNA was added 5 minutes later. The mixture was incubated at room temperature for 45 minutes then added to one well of a 6-well plate with 40% confluent 293T cells in DMEM with 10% FBS. The transfections were incubated at 37°C for 72 hours, then the supernatants were collected and centrifuged with 24,000 × g for 1 minute. The supernatants were aliquoted and stored at -80°C. The p24 antigen of the supernatants was analyzed by ELISA (Beckman Coulter, Miami, FL) and infectivity was determined by JC53-BL assay as described previously.

### Infection of SupT1 and virus replication

The viral supernatants with 1000IU infectivity were used to infect 1 × 10^6 ^SupT1 cells. The mixture of virus and SupT1 cells were incubated at 37°C for 4 hours, shaking every 30 minutes. The mixtures were then transferred to 25 cm^2 ^tissue culture flasks and the final volumes were adjusted to 10 mL by RPMI 1640 plus 15% FBS and 1% antibiotic-antimycotic.

The infected SupT1 cells were passaged at 1:4 every 3 days. When the cells were cleared because of syncytia, the fresh 1 × 10^6 ^SupT1 cells were added to all cultures. Every 7 days, 1 mL of cell culture was collected and centrifuged at 24,000 × g for 1 minute. The supernatant and cell pellets were store at -80°C for further analysis.

### DNA sequencing of viral U5-PBS region

High-molecular-Weight DNA (HWM) was extracted from infected SupT1 cell pellets by Wizard genomic DNA purification kit (Promega, Madison, WI) following the protocol. The fragment containing U5-PBS region was amplified from HMW by PCR with primers *EcoRI *(5'-CGGAATTCTCTCTCCTTCTAGCCTCCGCTAGTC-3') and *SphI *(5'-CCTTGAGCATGCGATCTACCACACACAAGGC-3'). The resulting PCR products were run on 1% agarose gel and the amplified fragments were cut and purified with the Qiagen Gel Purification kit (Qiagen, Valencia, CA). The sequence of purified DNA was determined by automated DNA sequencing with *EcoRI *as primer.
